# Long-Term Survival and Causes of Death After Diagnoses of Common Cancers in 3 Cohorts of US Health Professionals

**DOI:** 10.1093/jncics/pkac021

**Published:** 2022-03-08

**Authors:** En Cheng, Dong Hoon Lee, Rulla M Tamimi, Susan E Hankinson, Walter C Willett, Edward L Giovannucci, A Heather Eliassen, Meir J Stampfer, Lorelei A Mucci, Charles S Fuchs, Donna Spiegelman

**Affiliations:** 1 Department of Chronic Disease Epidemiology, Yale School of Public Health, New Haven, CT, USA; 2 Division of Research, Kaiser Permanente Northern California, Oakland, CA, USA; 3 Department of Nutrition, Harvard T.H. School of Public Health, Boston, MA, USA; 4 Department of Epidemiology, Harvard T.H. School of Public Health, Boston, MA, USA; 5 Channing Division of Network Medicine, Department of Medicine, Brigham and Women’s Hospital and Harvard Medical School, Boston, MA, USA; 6 Population Health Sciences, Weill Cornell Medicine, New York, NY, USA; 7 Division of Hematology and Medical Oncology, Department of Internal Medicine, Yale School of Medicine, New Haven, CT, USA; 8 Yale Cancer Center, Smilow Cancer Hospital, New Haven, CT, USA; 9 Department of Biostatistics, Yale School of Public Health, New Haven, CT, USA; 10 Center for Methods in Implementation and Prevention Science, Yale School of Public Health, New Haven, CT, USA

## Abstract

**Background:**

Few studies investigated long-term overall survival and causes of death among men and women diagnosed with most commonly occurring cancers.

**Methods:**

We estimated long-term (≥30-year) overall and cause-specific cumulative mortality for men diagnosed with prostate (n = 6873), lung and bronchus (n = 1290), colon and rectum (n = 1418), bladder (n = 1321), and melanoma (n = 2654) cancer in the Health Professionals Follow-up Study between 1986 and 2012 and women with breast (n = 18 280), lung and bronchus (n = 3963), colon and rectum (n = 3461), uterine corpus (n = 1641), and thyroid (n = 1103) cancer in the Nurses’ Health Study between 1976 and 2012 and Nurses’ Health Study II between 1989 and 2013.

**Results:**

We reported overall and cause-specific cumulative mortality of 30 years among men and 35 years among women. Among male cancer survivors, the 30-year cumulative cancer-specific mortality was 15.4% (95% confidence interval [CI] = 14.4% to 16.4%) for prostate, 83.5% (95% CI = 81.2% to 85.5%) for lung and bronchus, 37.0% (95% CI = 34.4% to 39.5%) for colon and rectum, 22.5% (95% CI = 20.0% to 25.0%) for urinary bladder, and 8.0% (95% CI = 6.9% to 9.1%) for melanoma. Among female cancer survivors, the 35-year cumulative cancer-specific mortality rate was 20.6% (95% CI = 19.7% to 21.6%) for breast, 83.5% (95% CI = 81.6% to 85.2%) for lung and bronchus, 39.6% (95% CI = 37.5% to 41.6%) for colon and rectum, 16.6% (95% CI = 14.7% to 18.6%) for uterine corpus, and 3.2% (95% CI = 2.1% to 4.3%) for thyroid. Except for lung cancer, most patients with common cancer were more likely to die from causes other than primary cancers. We observed 2 basic trends for cumulative cancer-specific mortality. The first is a sustained but nevertheless excess risk: Prostate or breast cancer-specific cumulative mortality continued to increase after diagnosis from 5 to 30 years or longer. The second is greatly diminished risk of index cancer-specific mortality following diagnosis 10 years or longer previously. For example, colorectal cancer–specific mortality increased by less than 4 percentage points between 10 and 30 or 35 years after diagnosis, and this finding also applied to lung, bladder, melanoma, uterine corpus, and thyroid cancer.

**Conclusions:**

Except for lung cancer, patients diagnosed with common cancers were more likely to die from causes other than primary cancers. Patients with lung, colorectal, bladder, melanoma, uterine corpus, or thyroid cancer surviving longer than 10 years after diagnosis are unlikely to die from that disease.

In 2020, an estimated 1 806 590 new cases of cancer were diagnosed in the United States (1). The 5 leading cancer types among men are prostate, lung and bronchus, colon and rectum, urinary bladder, and melanoma and among women are breast, lung and bronchus, colon and rectum, uterine corpus, and thyroid (1). These leading cancers in aggregate account for 56.9% of new cases in men and 61.8% in women (1); we designate them as common cancers in this article. These common cancers have a profound psychological impact on patients and their families (2), and they confer a large financial burden on patients and the health-care system (3-5).

Survival for common cancers except uterine corpus have markedly increased since 1975, likely because of advances in early screening and treatment, but the focus in reporting survival is usually 5-year survival (6–11). Although some studies investigated long-term (>10-year) overall survival (OS) after diagnoses of common cancer several decades ago (12–20), few studies have looked beyond 20 years, particularly in the United States. In addition, the problem of competing risks arises importantly in long-term survival studies (21), and it is not well understood how diseases other than the primary cancer may contribute to death after cancer diagnosis. In this article, we estimated long-term (15-year, 20-year, 25-year, and 30-year [plus 35-year for women]) cumulative OS and causes of death among men and women diagnosed with common cancers in the Health Professionals Follow-up Study (HPFS) (22), Nurses’ Health Study (NHS) (23), and NHS II (23).

## Methods

### Study Population and Design

HPFS, a prospective study established in 1986, enrolled 51 529 male health professionals aged 40 to 75 years. NHS, established in 1976, enrolled 121 700 female nurses aged 30 to 55 years, and NHS II enrolled 116 430 female nurses aged 25 to 42 years in 1989. NHS and NHS II were combined into 1 cohort for this analysis. Baseline and follow-up questionnaires were sent every 2 years to collect medical, lifestyle, and family history information; follow-up rates exceeded 90% in all 3 cohorts (23,24). The follow-up for diagnoses of common cancers was through 2012 for HPFS and NHS and 2013 for NHS II. The end of follow-up for death was set as 2018 for HPFS, 2016 for NHS, and 2017 for NHS II. For each patient with cancer, age at diagnosis was ascertained and categorized into 5-year intervals. The study protocol was approved by the institutional review boards of Yale University, the Brigham and Women’s Hospital, Harvard T.H. Chan School of Public Health, and those of participating registries, as required.

### Cancer Diagnosis

In every questionnaire, participants were asked if cancers had been diagnosed and, if yes, the diagnosis dates were registered. Permission was obtained to collect medical records and pathology reports. Physicians reviewed all medical records and pathology reports to confirm the cancer diagnoses. Regional tumor registries are regularly searched to confirm additional cancer diagnoses if participants were known to die from cancer but their medical records were inaccessible. We often learn of incident cancers from the families and the death follow-up process (see below).

### Ascertainment of Deaths and Causes of Deaths

We used reports of the participants’ families, state vital statistics records, the National Death Index, and the US Postal Service system to identify deaths and achieved 98% ascertainment by applying these methods (25). Once a death was reported, we attempted to contact the next of kin or other contact person, if necessary, to ascertain the cause of death and secure permission to obtain medical records. Following the *International Classification of Diseases, Eighth Revision* and *Ninth Revision* (26), all information was reviewed by physicians to determine the primary cause of death.

### Statistical Analysis

We used the Kaplan-Meier method to calculate cumulative all-cause mortality at 5, 10, 15, 20, 25, and 30 years (27). Additionally, we calculated 35-year cumulative mortality for women. Based on the method of Fine and Gray (28,29) for competing risks, we estimated cumulative cancer-specific mortality as the subdistribution cumulative mortality that quantifies the risk of death from a primary cancer in the presence of other diseases as competing events. This method hybridizes the ideas of traditional approach (the Kaplan-Meier method) and competing causal pathways and provides an estimate of risk of dying from primary cancer if all other causes were to be removed. If the absolute increase of cancer-specific cumulative mortality was less than 5 percentage points from 10 to 30 or 35 years after diagnosis, patients were considered unlikely to die from this type of cancer. All-cause and cancer-specific cumulative mortality for this analysis, strictly speaking, are not rates but probabilities (%) of death from any cause or the index cancer. Survival was calculated as 1 minus cumulative mortality.

To increase the generalizability of our survival estimates to the US White general population, we used the indirect standardization method to calculate standardized incidence ratios and standardized mortality ratios for common cancers (30), where the incidence and mortality rates (per 100 000 person-years) of the reference population were obtained from the National Cancer Institute Surveillance, Epidemiology, and End Results Program (SEER), 2012 to 2016 (30–32). We also applied the Ederer II method to calculate relative survival of primary cancers (33,34), defined as the ratio of the observed survival of cancer patients to the expected survival of a comparable US White general-population sample matched on age, sex, race, and calendar year from all causes, including from the primary cancer (33,35). This quantity provides information about the extent to which cancer survivor differs appreciably from OS in the White general population: Relative survival greater than 100.0% indicates that OS of cancer patients was higher than the White general population. Expected survival was calculated from the National Center for Health Statistics life tables (36).

To generalize our findings in cumulative cancer-specific mortality to the US White general population, we applied the method of Fine and Gray to the SEER 18 Regs Research Database, November 2018 Submission (1975-2016) (28,29,37). In addition, we compared important diet and lifestyle characteristics between our cohorts and US White general population (38,39).

Point estimates were presented with 95% CIs. Analyses were performed using SAS statistical software, version 9.4 for UNIX (SAS Institute Inc), and R, version 3.6.0 for UNIX (R Foundation for Statistical Computing).

## Results

### Patient Characteristics

Age at diagnosis and death among men and women diagnosed with common cancers are presented in Tables 1 and 2. Among men, we confirmed 6873 cases of prostate cancer (median age at diagnosis = 70.3 years), 1290 cases of lung and bronchus cancer (median age at diagnosis = 74.2 years), 1418 cases of colorectal cancer (median age at diagnosis = 71.7 years), 1321 cases of urinary bladder cancer (median age at diagnosis = 73.9 years), and 2654 cases of melanoma (median age at diagnosis = 70.9 years). Among women, we confirmed 18 280 cases of breast cancer (median age at diagnosis = 59.3 years), 3963 cases of lung and bronchus cancer (median age at diagnosis = 69.2 years), 3461 cases of colorectal cancer (median age at diagnosis = 65.9 years), 1641 cases of uterine corpus cancer (median age at diagnosis = 62.8 years), and 1103 cases of thyroid cancer (median age at diagnosis = 53.4 years).

**Table 1.** Age at diagnosis and death among men (1986-2018) diagnosed with common cancers^a^

Characteristics	Men				
	Prostate	Lung and bronchus	Colon and rectum	Urinary bladder	Melanoma
	(n = 6873)	(n = 1290)	(n = 1418)	(n = 1321)	(n = 2654)
Age at diagnosis, median (IQR), y	70.3 (65.1-75.3)	74.2 (67.4-80.1)	71.7 (65.0-77.9)	73.9 (67.0-80.0)	70.9 (63.6-77.8)
Age at diagnosis, No. (%)					
40-44 y	—	1 (0.1)	3 (0.2)	2 (0.2)	22 (0.8)
45-49 y	19 (0.3)	7 (0.5)	24 (1.7)	11 (0.8)	60 (2.3)
50-54 y	109 (1.6)	24 (1.9)	53 (3.7)	23 (1.7)	124 (4.7)
55-59 y	463 (6.7)	74 (5.7)	86 (6.1)	63 (4.8)	214 (8.1)
60-64 y	1064 (15.5)	121 (9.4)	181 (12.8)	138 (10.4)	343 (12.9)
65-69 y	1714 (24.9)	206 (16.0)	271 (19.1)	210 (15.9)	466 (17.6)
70-74 y	1682 (24.5)	251 (19.5)	291 (20.5)	283 (21.4)	499 (18.8)
75-79 y	1203 (17.5)	273 (21.2)	248 (17.5)	255 (19.3)	437 (16.5)
≥80 y	619 (9.0)	333 (25.8)	261 (18.4)	336 (25.4)	489 (18.4)
Total deaths, No. (%)	3991 (58.1)	1244 (96.4)	1132 (79.8)	906 (68.6)	1516 (57.1)
Deaths from the primary cancer, No. (%)	883 (12.8)	1067 (82.7)	516 (36.4)	279 (21.1)	197 (7.4)

a IQR = interquartile range.

**Table 2.** Age at diagnosis and death among women (1976-2017) diagnosed with common cancers^a^

Characteristics	Women				
	Breast	Lung and bronchus	Colon and rectum	Uterine corpus	Thyroid
	(n = 18 280)	(n = 3963)	(n = 3461)	(n = 1641)	(n = 1103)
Age at diagnosis, median (IQR), y	59.3 (51.2-67.8)	69.2 (61.8-75.4)	65.9 (57.7-73.6)	62.8 (56.2-69.8)	53.4 (46.1-61.7)
Age at diagnosis, No. (%)					
25-29 y	8 (0.0)	—	—	—	5 (0.5)
30-34 y	82 (0.4)	2 (0.1)	5 (0.1)	3 (0.2)	23 (2.1)
35-39 y	410 (2.2)	12 (0.3)	33 (1.0)	8 (0.5)	81 (7.3)
40-44 y	1198 (6.6)	52 (1.3)	69 (2.0)	31 (1.9)	133 (12.1)
45-49 y	2224 (12.2)	105 (2.6)	181 (5.2)	96 (5.9)	167 (15.1)
50-54 y	2749 (15.0)	238 (6.0)	342 (9.9)	214 (13.0)	212 (19.2)
55-59 y	2860 (15.6)	397 (10.0)	460 (13.3)	291 (17.7)	166 (15.0)
60-64 y	2771 (15.2)	557 (14.1)	540 (15.6)	327 (19.9)	109 (9.9)
65-69 y	2405 (13.2)	742 (18.7)	547 (15.8)	270 (16.5)	93 (8.4)
70-74 y	1775 (9.7)	798 (20.1)	561 (16.2)	215 (13.1)	58 (5.3)
75-79 y	1198 (6.6)	634 (16.0)	414 (12.0)	123 (7.5)	40 (3.6)
≥80 y	600 (3.3)	426 (10.7)	309 (8.9)	63 (3.8)	16 (1.5)
Total deaths, No. (%)	6431 (35.2)	3534 (89.2)	2101 (60.7)	752 (45.8)	170 (15.4)
Deaths from the primary cancer, No. (%)	2777 (15.2)	3158 (79.7)	1277 (36.9)	258 (15.7)	32 (2.9)

a IQR = interquartile range.

### All-Cause and Cancer-Specific Cumulative Mortality

All-cause and cancer-specific cumulative mortality among men and women diagnosed with common cancers is presented in Table 3.

**Table 3.** All-cause and cancer-specific cumulative mortality of men (1986-2018) and women (1976-2017) diagnosed with common cancers

Cumulative mortality	Cumulative mortality, % (95% CI)						
	5-y	10-y	15-y	20-y	25-y	30-y	35-y
All cause^a^							
Men							
Prostate	11.9 (11.2-12.7)	28.4 (27.3-29.5)	46.5 (45.2-47.7)	63.8 (62.3-65.1)	81.0 (79.4-82.6)	91.4 (88.6-93.6)	—
Lung and bronchus	84.8 (82.7-86.6)	91.7 (90.1-93.1)	95.9 (94.5-96.9)	97.3 (96.1-98.2)	98.4 (97.2-99.1)	98.4 (97.2-99.1)	—
Colon and rectum	39.4 (36.8-41.9)	56.2 (53.5-58.7)	67.6 (65.0-70.0)	78.2 (75.7-80.5)	86.9 (84.4-88.9)	93.2 (90.3-95.3)	—
Urinary bladder	28.4 (25.9-30.8)	47.7 (44.9-50.3)	64.9 (61.9-67.7)	75.6 (72.6-78.3)	83.9 (80.7-86.6)	90.1 (85.8-93.1)	—
Melanoma	15.6 (14.2-17.0)	33.5 (31.7-35.4)	48.9 (46.7-50.9)	61.6 (59.3-63.8)	71.5 (68.9-73.8)	81.2 (78.2-83.8)	—
Women							
Breast	10.7 (10.2-11.1)	20.2 (19.6-20.8)	29.5 (28.8-30.2)	38.9 (38.0-39.7)	48.6 (47.5-49.7)	58.9 (57.5-60.3)	68.9 (67.0-70.6)
Lung and bronchus	77.7 (76.4-78.9)	85.7 (84.5-86.8)	90.9 (89.8-91.9)	93.6 (92.5-94.5)	95.7 (94.5-96.6)	96.8 (95.6-97.7)	97.8 (96.4-98.6)
Colon and rectum	36.7 (35.1-38.3)	47.7 (46.0-49.4)	56.0 (54.1-57.7)	63.4 (61.4-65.3)	71.9 (69.7-73.9)	79.2 (76.6-81.5)	86.5 (82.9-89.3)
Uterine corpus	17.9 (16.0-19.7)	26.2 (24.0-28.4)	35.7 (33.1-38.1)	46.0 (43.1-48.8)	57.4 (53.9-60.7)	71.1 (66.8-74.9)	79.5 (74.0-83.9)
Thyroid	3.6 (2.5-4.7)	8.0 (6.3-9.7)	12.2 (9.9-14.4)	19.1 (15.9-22.2)	22.8 (18.9-26.4)	29.6 (24.0-34.8)	42.6 (33.7-50.4)
Cancer specific^b^							
Men							
Prostate	5.4 (4.9-5.9)	9.1 (8.5-9.8)	11.8 (11.0-12.6)	13.8 (12.9-14.7)	15.2 (14.2-16.2)	15.4 (14.4-16.4)	—
Lung and bronchus	78.1 (75.8-80.3)	81.4 (79.1-83.4)	82.9 (80.7-84.8)	83.0 (80.8-85.0)	83.5 (81.2-85.5)	83.5 (81.2-85.5)	—
Colon and rectum	31.0 (28.6-33.4)	35.2 (32.7-37.7)	36.1 (33.6-38.6)	36.4 (33.9-38.9)	37.0 (34.4-39.5)	37.0 (34.4-39.5)	—
Urinary bladder	16.4 (14.3-18.3)	19.6 (17.4-21.7)	21.5 (19.2-23.8)	21.9 (19.5-24.2)	22.5 (20.0-25.0)	22.5 (20.0-25.0)	—
Melanoma	5.8 (4.9-6.6)	6.8 (5.9-7.8)	7.3 (6.3-8.3)	7.6 (6.5-8.6)	8.0 (6.9-9.1)	8.0 (6.9-9.1)	—
Women							
Breast	7.7 (7.3-8.1)	12.2 (11.7-12.7)	15.2 (14.6-15.7)	17.1 (16.5-17.7)	18.7 (18.0-19.4)	19.9 (19.0-20.7)	20.6 (19.7-21.6)
Lung and bronchus	74.5 (73.1-75.8)	79.0 (77.6-80.2)	81.1 (79.7-82.4)	82.0 (80.6-83.3)	82.5 (81.0-83.9)	83.0 (81.4-84.5)	83.5 (81.6-85.2)
Colon and rectum	31.8 (30.2-33.3)	35.8 (34.2-37.4)	37.4 (35.7-39.0)	37.9 (36.2-39.6)	38.6 (36.8-40.3)	39.2 (37.3-41.0)	39.6 (37.5-41.6)
Uterine corpus	13.2 (11.5-14.8)	15.0 (13.5-16.8)	15.7 (13.9-17.5)	16.0 (14.2-17.8)	16.3 (14.5-18.2)	16.6 (14.7-18.6)	16.6 (14.7-18.6)
Thyroid	2.1 (1.2-2.9)	2.8 (1.8-3.9)	3.0 (1.9-4.0)	3.2 (2.1-4.3)	3.2 (2.1-4.3)	3.2 (2.1-4.3)	3.2 (2.1-4.3)

a All-cause cumulative mortality was calculated by the Kaplan-Meier method. CI = confidence interval.

b Cancer-specific cumulative mortality was calculated by the method of Fine and Gray.

**Figure 1.**
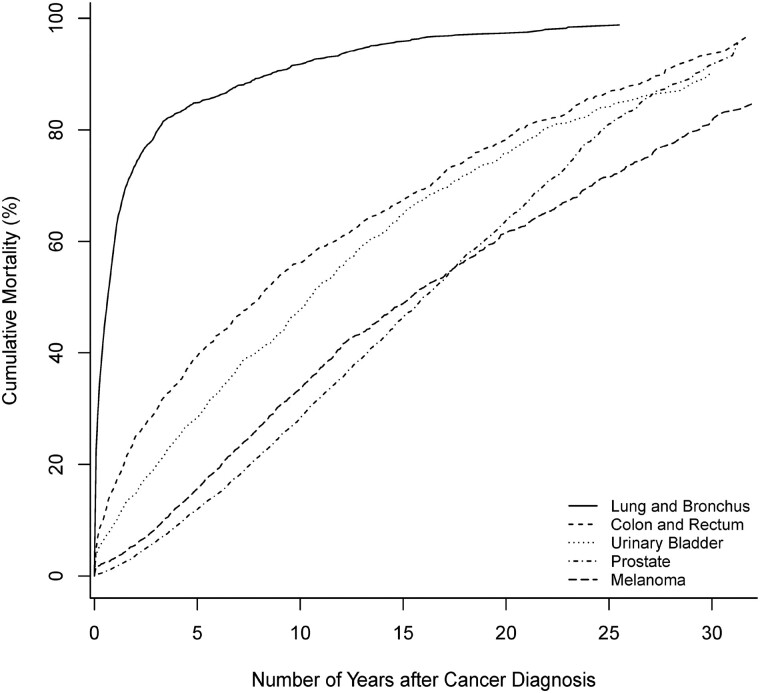
All-cause cumulative mortality (%) of men diagnosed with common cancers, 1986-2018.

**Figure 2.**
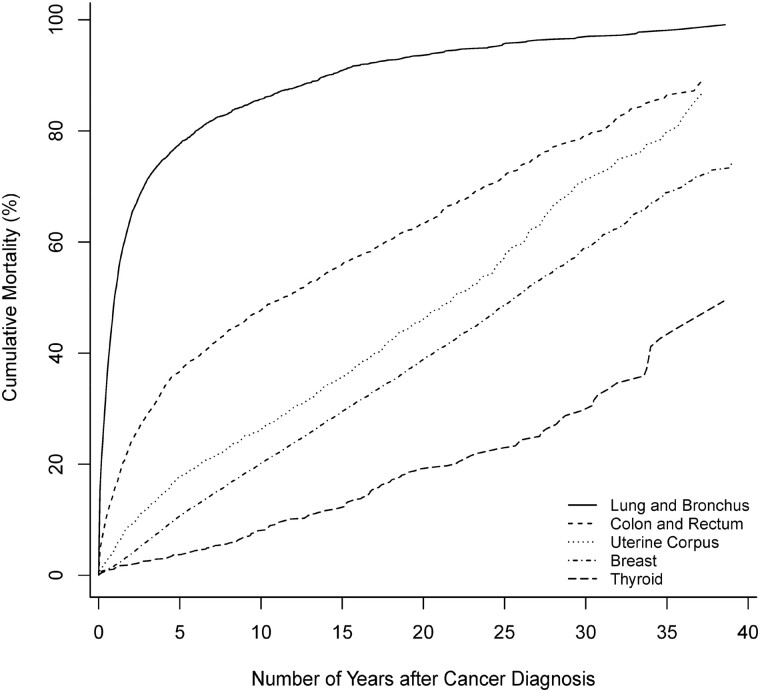
All-cause cumulative mortality (%) of women diagnosed with common cancers, 1976-2017.

Among men and women, all-cause cumulative mortality of common cancers over the 30 or 35 years since diagnosis increased with increasing years from cancer diagnosis (Figures 1 and 2). For lung cancer, among men, the all-cause cumulative mortality increased rapidly within 5 and 10 years after cancer diagnosis to 84.8%, (95% CI = 82.7% to 86.6%) and 91.7% (95% CI = 90.1% to 93.1%); among women, it increased to 77.7% (95% CI = 76.4% to 78.9%) and 85.7% (95% CI = 84.5% to 86.8%). Regarding lung cancer–specific cumulative mortality, of men, although 83.5% (95% CI = 81.2% to 85.5%) died from lung cancer by 30 years, 78.1% (95% CI = 75.8% to 80.3%) died from lung cancer within the first 5 years, and 81.4% (95% CI = 79.1% to 83.4%) died within 10 years. Among women, although 83.5% (95% CI = 81.6% to 85.2%) died from lung cancer by 35 years, 74.5% (95% CI = 73.1% to 75.8%) died from lung cancer within the first 5 years, and 79.0% (95% CI = 77.6% to 80.2%) died within 10 years.

**Figure 3.**
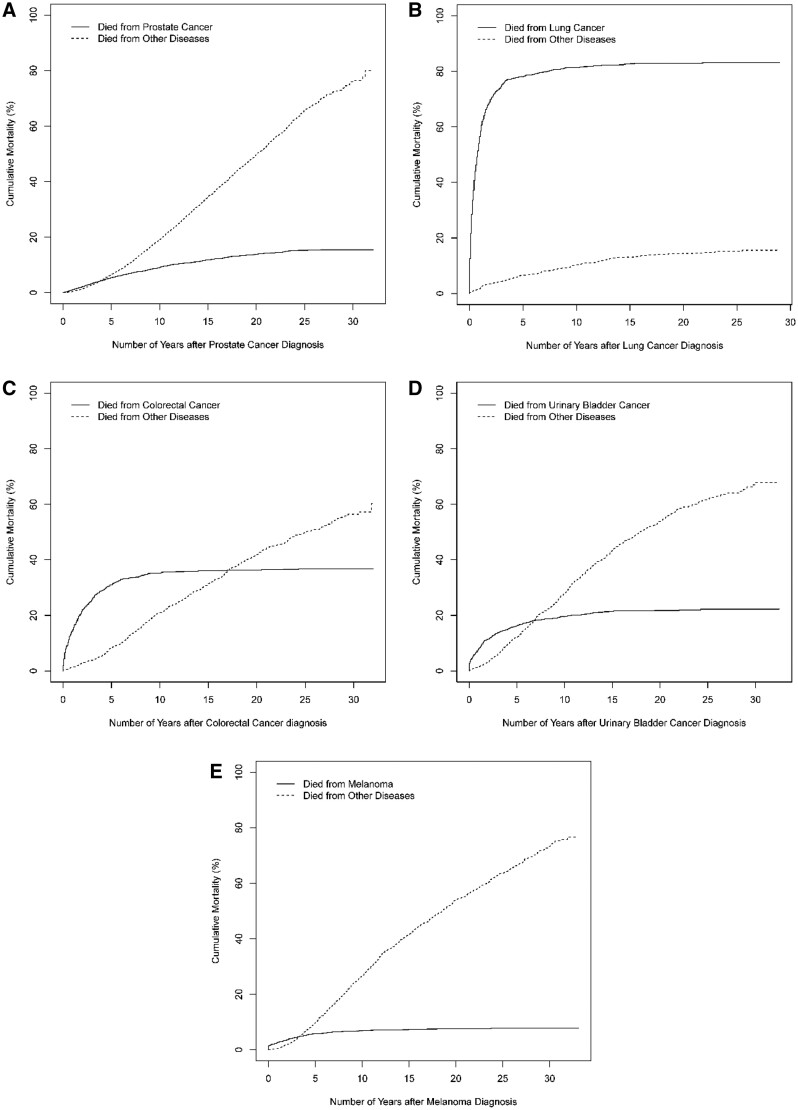
Cancer-specific cumulative mortality (%) of men diagnosed with common cancers, 1986-2018. Data for cancers of the **A**) prostate, **B**) lung, **C**) colorectal, **D**) urinary bladder, and **E**) melanoma are shown.

**Figure 4.**
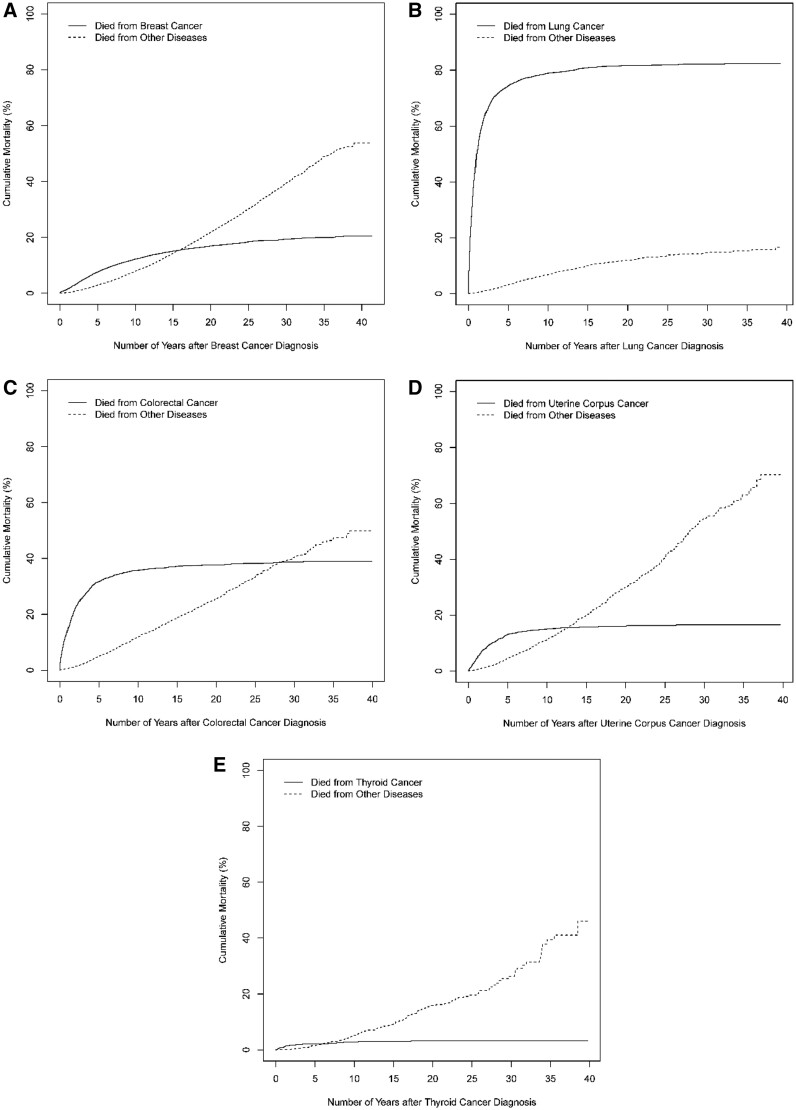
Cancer-specific cumulative mortality (%) of women diagnosed with common cancers, 1976-2017. Data for cancers of the **A**) breast, **B**) lung, **C**) colorectal, **D**) uterine corpus, and **E**) thyroid are shown.

Of patients with prostate cancer, the cancer-specific cumulative morality increased to 15.4% (95% CI = 14.4% to 16.4%) over 30 years (Figure 3); among female patients with breast cancer, such cumulative mortality increased to 20.6% (95% CI = 19.7% to 21.6%) over 35 years (Figure 4). The primary cancers (prostate or breast cancer), however, contributed much less to overall mortality in these patients compared with other diseases. For lung, colorectal, urinary bladder, melanoma, uterine corpus, and thyroid cancer, most deaths from the primary cancer occurred within 10 years following diagnosis, such that the cancer-specific cumulative mortality from these primary cancers increased minimally after 10 years while the cumulative mortality from other causes increased progressively. The top 3 noncancer causes of death are presented in Supplementary Tables 1 and 2 (available online); cardiovascular disease was the most common contributor to noncancer causes of death.

Although year 10 is a commonly used clinical landmark for cancer progression, this empirically selected point may not match the year estimated to correspond to the change in the slope of the cancer-specific mortality curves. Thus, for lung, colorectal, urinary bladder, melanoma, uterine corpus, and thyroid cancer, we further quantified inflection points and compared slopes of inflection points and year 10 (Supplementary Table 3, available online) (40–43). For these cancers, the slope after year 10 was smaller than the slope before year 10. Although the cancer-specific mortality risk continued to increase after 10 years, the magnitude of this increase greatly diminished. These findings supported year 10 as a clinical landmark that could be useful for clinicians and patients managing postdiagnostic survival expectations.

### Standardized Incidence Ratio, Standardized Mortality Ratio, and Relative Survival

Compared with cancer incidence rates of the White general population, the incidence rates of HPFS were 1) higher for prostate and melanoma, 2) somewhat lower but similar for colon and rectum, and 3) lower for lung and bronchus and urinary bladder. This trend also applies to the findings of mortality rates, but the mortality rate of melanoma in HPFS was similar to that of the White general population (Supplementary Table 4, available online). For women in NHS and NHS II, the incidence rates were 1) somewhat higher for breast; 2) somewhat lower but similar for colon and rectum; and 3) lower for lung and bronchus, uterine corpus, and thyroid, while mortality rates were 1) somewhat higher but similar for breast, colon, and rectum; 2) somewhat lower but similar for uterine corpus and thyroid; and 3) lower for lung and bronchus (Supplementary Table 5, available online).

The relative survival results are presented in Supplementary Figures 1 and 2 and Supplementary Tables 6 and 7 (available online). Among men, after cancer diagnosis of prostate, urinary bladder, and melanoma, relative survival increased away from more than 100.0% as time from diagnosis increased. The relative survival of lung cancer decreased to 15.5% within the first 10 years and remained around 11.0% after that. For colorectal cancer, it decreased to 81.8% within the first 10 years and increased after that. Among women, after cancer diagnosis of breast, uterine corpus, and thyroid, relative survival increased and hovered around 100.0%. The relative survival for lung cancer decreased to 19.3% within the first 10 years; after that, it decreased more. The relative survival for colorectal cancer decreased to 69.3% within the first 10 years and showed an increasing trend after that.

### Generalizability of Cumulative Cancer-Specific Mortality

Similar to findings of cumulative cancer-specific mortality observed in 3 cohorts, in the SEER database, we found that 1) most patients with lung cancer died from lung cancer within 10 years of diagnosis; 2) cancer-specific cumulative morality of prostate and breast cancer continued to increase through 30- or 35-year follow-up; and 3) cancer-specific cumulative mortality from lung, colorectal, urinary bladder, melanoma, uterine corpus, and thyroid cancer increased minimally after 10 years (Supplementary Figures 3 and 4, available online). Compared with the US White general population, cohort participants were less likely to be current smokers or drink and were more likely to meet physical activity guidelines, have higher scores of diet quality, and maintain lower body mass index (Supplementary Table 8, available online).

## Discussion

In this descriptive study, we reported long-term cumulative OS and causes of death among men and women diagnosed with common cancers. Most patients with lung and bronchus cancer died within 10 years of diagnosis from their primary cancers. We observed 2 basic trends for cumulative cancer-specific mortality. The first is sustained but low excess risk after cancer diagnosis, and it applied to prostate and breast cancer. The second is greatly diminished risk of the index cancer-specific mortality 10 years or more following diagnosis, and it applied to lung, colorectal, urinary bladder, melanoma, uterine corpus, and thyroid cancer.

Several decades ago, data on long-term overall survival from common cancers in the United States and European countries, including the United Kingdom, Sweden, and the Netherlands, were published (12–20). This study updates this long-term OS and is the first to present 30-year and 35-year OS and cause-specific survival. In line with previous studies, all-cause cumulative mortality of common cancers increased after cancer diagnosis, and more than half of patients with lung cancer died within the first 5 years of cancer diagnosis; the majority died within 10 years after cancer diagnosis (12,13,16–20). Although 2 UK breast cancer studies conducted in the 1950s reported 5-year OS rates of 33.0% and 48.1% (14,15), substantial progress has been made, with a 89.3% 5-year OS seen here, likely reflecting a lack of early detection and access to effective treatment options during that earlier time. We acknowledge that, in addition to cancer types, age at diagnosis is a key factor affecting overall long-term (>10-year) survival (44). Given that few studies reported OS beyond 20 years, particularly in the United States, however, these data may help clinicians and their patients understand long-term cancer prognosis and evaluate lifelong survival expectations.

Several studies investigated causes of death within 15 years of cancer diagnosis among prostate and breast cancer (45–50). Consistent with previous studies, we found that other diseases (especially cardiovascular disease) contributed importantly to mortality among survivors of breast and prostate cancer; additionally, we reported causes of death 15 years and more following cancer diagnoses among patients with breast, prostate, and other common cancers. Cardiovascular disease, reported as the most common cause of noncancer death, may be explained by cardiotoxicity being a recognized adverse event of common cancer treatment, such as cytotoxic chemotherapy, targeted therapy, hormone therapy, and radiation therapy (51–53). Except for lung cancer, causes of death contributions from the primary cancers lagged behind other diseases, which may indicate expansion of early detection, preventative lifestyle interventions, and improved treatment for these cancers (48,49,54). Other independent comorbidities should receive sufficient attention from oncologists while treating primary cancers. Particularly for colon and rectum, urinary bladder, melanoma, uterine corpus, and melanoma cancers, for patients surviving more than 10 years beyond their cancer diagnosis, they were essentially cured of the primary cancers, and clinicians should look more closely at other diseases patients may have. For lung and bronchus cancer, rates of death from the primary cancers far exceeded death from other diseases, indicating the continued absence of breakthroughs in prognosis (12,54).

Because patients in 3 cohorts were mostly White health professionals, they may have different prognosis factors for cancer progression and higher socioeconomic status than the White general population (23,55), and we calculated standardized incidence ratio, standardized mortality ratio, and relative survival to address, at least in part, concerns about the generalizability of our findings. Compared with the US White general population, cohort participants were more likely to eat healthier diets, smoke less, drink less, maintain lower body mass index, and meet physical activity guidelines (38); thus, they may have lower rates of smoking-related diseases and cardiovascular diseases. The lower incidence and mortality rates from lung cancer likely reflect these healthier lifestyles. Moreover, cohort participants may have better access to cancer screening and treatment as well as better healthcare quality, which then may be reflected by higher incidence rates of prostate cancer and melanoma and high relative survival rates from most cancers except lung cancer. The low relative survival rates after lung cancer diagnosis, which remained fairly stable after 10 years, is consistent with the fact that these primary cancers were major causes of death, especially within the first 10 years. In contrast, favorable relative survival from other common cancers may encourage patients to look beyond the primary cancer diagnosis and be alert for comorbidities.

The strengths of our study include its prospective design, large sample sizes, high rates of follow-up, reliable confirmation of cancer diagnosis, and accurate ascertainment of death. This study has certain limitations, however. First, within each cancer, survival and causes of death could be different by stage or other clinical features, such as molecular subtypes (56). For example, 5-year survival for colon cancer was greater than 90% for stage I disease but less than 10% for stage IV disease (57); 80% of breast cancers are estrogen receptor positive (58), the survival rate for which is higher than for estrogen receptor–negative cancer (59,60). Such uncontrolled features at both the person and tumor levels may temper our conclusions, and future studies could explore the differences in long-term survival and causes of death after cancer diagnosis by adjusting for these features.

Second, because the cohorts collected limited information about treatment and these common cancers were diagnosed as early as 1986 among men and 1976 among women, results may not reflect the most recent progress in cancer treatment, particularly underestimating long-term survival and cause-specific mortality by other diseases. Our findings may still help clinicians and their patients evaluate lifelong survival expectations, however, and plan long-term interventions for primary cancers and other diseases as causes of death. In addition, long-term survival standardized to the age at diagnosis distribution of US cancer survivors in general may help direct public health resources. Although it may limit generalizability that patients were mostly White healthcare professionals, our findings are broadly consistent with previous studies and uniquely provide long-term survival rates and causes of death among US cancer survivors previously unavailable. In addition, we analyzed SEER data to strengthen the generalizability of our findings. To the extent that cohort participants have healthier lifestyles and better access to health care, the findings here suggest best-case scenarios of what can be aspired to more broadly.

We observed 2 general trends in long-term survival from primary cancers among male and female health professionals diagnosed with common cancers. Patients diagnosed with lung and bronchus cancer had poor short- and long-term survival rates and mostly died from their primary cancer. Patients diagnosed with the other common cancers had better survival after cancer diagnosis, and other diseases contributed more than the primary cancers to death in the long term. For prostate cancer and breast cancer in women, in addition to primarily focusing on other diseases, clinicians and patients should also monitor the index cancer for treatment. Among men diagnosed with lung, colorectal, urinary bladder, and melanoma cancer and women diagnosed with lung, colorectal, uterine corpus, and thyroid cancer, other diseases should receive more attention in the treatment of survivors beyond 10 years. Our findings provide important insight into how cancer survivors and their clinicians should best manage their long-term health care for the primary cancers and other diseases. Future studies should investigate long-term survival and nonprimary-cancer causes of death among patients diagnosed with common cancers by stage, treatment, time trends, molecular subtype, and other clinical and lifestyle characteristics. Future studies could also focus on the deadliest cancers given that most common cancers are not necessarily the deadliest.

## Funding

This study was supported, in part, by grants from the National Institutes of Health: UM1 CA186107, U01 CA176726, U01 CA167552, and P01 CA87969.

## Notes

**Role of the funder:** The funder had no role in the design of the study; the collection, analysis, and interpretation of the data; the writing of the manuscript; or the decision to submit the manuscript for publication.

**Disclosures**: The authors have no conflicts of interest to disclose.

**Acknowledgements:** We would like to thank the participants and staff of the Healthy Professionals Follow-up Study, Nurses’ Health Study, and Nurses’ Health Study II for their valuable contributions as well as the following state cancer registries for their help: AL, AZ, AR, CA, CO, CT, DE, FL, GA, ID, IL, IN, IA, KY, LA, ME, MD, MA, MI, NE, NH, NJ, NY, NC, ND, OH, OK, OR, PA, RI, SC, TN, TX, VA, WA, WY. The authors assume full responsibility for analyses and interpretation of these data.

**Disclaimer:** The content is solely the responsibility of the authors and does not necessarily represent the official views of the National Institutes of Health.

**Author contributions:** Conceptualization: EC, DS. Data curation: WCW, AHE, MJS, LAM. Formal analysis: EC, DS. Funding acquisition: WCW, AHE, MJS, LAM, CSF, DS. Investigation: WCW, ELG, AHE, MJS, LAM, DS. Methodology: EC, DS. Project administration: WCW, AHE, MJS, LAM. Resources: WCW, ELG, AHE, MJS, LAM, DS. Software: EC, DHL, DS. Supervision: EC, DS. Visualization: EC, DS. Writing (original draft); EC, DS. Writing (review and editing): All authors.

**Prior presentations**: This manuscript was presented as an abstract at the 2020 ASCO Annual Meeting.

## Data Availability

The data underlying this article were accessed from Channing Division of Network Medicine Cohorts [https://www.brighamandwomens.org/research/departments/channing-division-of-network-medicine/cohorts]. The derived data generated in this research will be shared on reasonable request to the corresponding author and primary investigators of Channing Division of Network Medicine Cohorts.

## Supplementary Material

pkac021_Supplementary_Data
