# Supplementary material for: Long-Term Survival and Causes of Death After Diagnoses of Common Cancers in 3 Cohorts of US Health Professionals
Source: JNCI Cancer Spectr. 2022 Mar 8;6(2):pkac021. doi: 10.1093/jncics/pkac021 (PMC8973409; doi:10.1093/jncics/pkac021)

SUPPLEMENTARY MATERIALS

Supplementary Tables

Supplementary Table 1. Top Three Non-Cancer Causes of Death among Men (1986 - 2018) Diagnosed with Common Cancers

Ranking	Prostate	Lung and Bronchus	Colon and Rectum	Urinary Bladder	Melanoma
1	Cardiovascular disease	Cardiovascular disease	Cardiovascular disease	Cardiovascular disease	Cardiovascular disease
2	Respiratory disease	Respiratory disease	Respiratory disease	Respiratory disease	Hereditary and familial diseases of nervous system
3	Senile and presenile dementia	Accidental Falls	Hereditary and familial diseases of nervous system	Hereditary and familial diseases of nervous system	Respiratory disease

Supplementary Table 2. Top Three Non-Cancer Causes of Death among Women (1976 - 2017) Diagnosed with Common Cancers

Ranking	Breast	Lung and Bronchus	Colon and Rectum	Uterine Corpus	Thyroid
1	Cardiovascular disease	Cardiovascular disease	Cardiovascular disease	Cardiovascular disease	Cardiovascular disease
2	Senile and presenile dementia	Respiratory disease	Respiratory disease	Senile and presenile dementia	Senile and presenile dementia
3	Respiratory disease	Senile and presenile dementia	Senile and presenile dementia	Respiratory disease	Respiratory disease

Supplementary Table 3. Slopes of Inflection Points and Year 10 for Colorectal, Urinary Bladder, Melanoma, Uterine Corpus, and Thyroid Cancer

Cancer	Inflection Point, years	*P*[a]	Percentage Point Increase in Cumulative Mortality Proportion per 1 Year after Diagnosis		
			Slope before Inflection Point /Slope before Year 10 (Percentage Point Increase per Year)	Slope after Inflection Point /Slope after Year 10 (Percentage Point Increase per Year)	Ratios (after vs. before) for Inflection Point/Year 10
Men					
Lung and Bronchus	15.4	<0.001	0.27/0.52	0.06/0.08	0.22/0.15
Colon and Rectum	10.3	0.16	1.30/2.74	0.09/0.12	0.07/0.04
Urinary Bladder	14.7	<0.001	0.40/0.56	0.09/0.20	0.23/0.36
Melanoma	18.0	<0.001	0.08/0.22	0.06/0.08	0.75/0.36
Women					
Lung and Bronchus	16.1	<0.001	0.45/0.49	0.12/0.23	0.27/0.47
Colon and Rectum	15.2	<0.001	0.29/0.46	0.13/0.18	0.45/0.39
Uterine Corpus	11.8	<0.001	0.25/0.45	0.08/0.10	0.32/0.22
Thyroid	17.5	1.00	0.04/0.11	0/0.04	0/0.36

[a] *P*-value was calculated using segmented regression model (2-sided).

Supplementary Table 4. Indirect Standardization of Incidence and Mortality Rates (1986-2018) of Common Cancers among Men using SEER Incidence and Mortality Rates (White Population), 2012-2016[a]

Cancer	Incidence Rate (per 100,000 person-years)			Mortality Rate (per 100,000 person-years)		
	HPFS	SEER	SIR	HPFS	SEER	SMR
Prostate	634.8	410.9	1.5	74.4	55.4	1.3
Lung and bronchus	93.6	242.2	0.4	79.3	203.5	0.4
Colon and rectum	105.0	132.1	0.8	43.7	53.0	0.8
Urinary bladder	87.2	139.3	0.6	20.6	26.9	0.8
Melanoma	181.2	114.6	1.6	14.8	14.7	1.0

[a] SEER= Surveillance, Epidemiology, and End Results; HPFS= Health Professionals Follow-up Study; SIR= Standardized Incidence Ratio; SMR= Standardized Mortality Ratio.

Supplementary Table 5. Indirect Standardization of Incidence and Mortality Rates of Common Cancers (1976-2017) among Women using SEER Incidence and Mortality Rates (White Population), 2012-2016[a]

Cancer	Incidence Rate (per 100,000 person-years)			Mortality Rate (per 100,000 person-years)		
	NHS and NHS II	SEER	SIR	NHS and NHS II	SEER	SMR
Breast	275.6	259.4	1.1	44.1	35.6	1.2
Lung and bronchus	54.1	98.5	0.5	47.0	67.1	0.7
Colon and rectum	45.6	56.7	0.8	19.3	18.1	1.1
Uterine corpus	21.3	58.1	0.4	6.8	7.9	0.9
Thyroid	14.8	39.6	0.4	0.6	0.7	0.8

[a] SEER= Surveillance, Epidemiology, and End Results; NHS= Nurses' Health Study; SIR= Standardized Incidence Ratio; SMR= Standardized Mortality Ratio.

Supplementary Table 6. Relative Survival % (95% CI) of Common Cancers among Men Compared to the U.S. White General Population Matched on Age, Sex, Race and Calendar Year

Year	Prostate	Lung and Bronchus	Colon and Rectum	Urinary Bladder	Melanoma
5-year	111.3 (110.3, 112.3)	20.0 (17.4, 22.6)	80.1 (76.7, 83.4)	98.0 (94.7, 101.3)	110.8 (109.0, 112.6)
10-year	124.0 (122.2, 125.9)	15.5 (12.7, 18.3)	81.8 (77.0, 86.7)	104.8 (99.4, 110.3)	121.4 (118.1, 124.7)
15-year	141.2 (137.9, 144.5)	11.4 (8.2, 14.5)	91.0 (84.0, 97.9)	108.7 (100.0, 117.3)	133.9 (128.6, 139.2)
20-year	166.7 (160.5, 172.9)	10.9 (6.9, 15.0)	97.2 (86.8, 107.6)	121.7 (107.7, 135.7)	149.3 (140.9, 157.7)
25-year	181.8 (167.9, 195.6)	10.8 (4.9, 16.7)	100.0 (83.8, 116.3)	139.9 (115.4, 164.3)	168.2 (154.6, 181.8)
30-year	186.7 (139.2, 234.2)	11.4 (4.3, 18.5)	76.2 (50.1, 102.3)	178.5 (133.0, 224.0)	166.8 (142.4, 191.2)

[a] CI = confidence interval.

Supplementary Table 7. Relative Survival % (95% CI) of Common Cancers among Women Compared to the U.S. White General Population Matched on Age, Sex, Race and Calendar Year[a]

Year	Breast	Lung and Bronchus	Colon and Rectum	Uterine Corpus	Thyroid
5-year	96.5 (96.0, 97.0)	25.5 (24.0, 27.0)	71.4 (69.6, 73.2)	89.6 (87.6, 91.6)	100.9 (99.7, 102.1)
10-year	95.5 (94.8, 96.3)	19.3 (17.8, 20.9)	69.3 (67.0, 71.5)	90.6 (87.9, 93.2)	102.5 (100.7, 104.4)
15-year	96.3 (95.3, 97.3)	14.9 (13.2, 16.6)	71.0 (68.2, 73.9)	92.3 (88.8, 95.8)	105.0 (102.4, 107.7)
20-year	98.1 (96.7, 99.5)	12.6 (10.6, 14.5)	74.3 (70.6, 78.1)	93.9 (89.0, 98.7)	104.7 (100.7, 108.7)
25-year	100.6 (98.6, 102.6)	10.3 (8.0, 12.6)	75.9 (70, 5, 81.3)	96.3 (88.8, 103.7)	110.7 (105.4, 115.9)
30-year	103.0 (99.7, 106.2)	9.0 (6.1, 12.0)	79.7 (71.1, 88.3)	89.4 (77.4, 101.3)	118.8 (109.9, 127.7)
35-year	105.1 (99.5, 110.7)	7.1 (3.1, 11.0)	73.6 (57.6, 89.5)	93.9 (71.8, 115.9)	117.2 (100.1, 134.2)

[a] CI = confidence interval.

Supplementary Table 8. Comparison of Lifestyle Factors in the Three Cohorts and in the US White Population in 2010[a]

	Men		Women	
Characteristics	HPFS	US White Population	NHS and NHS II	US White Population
Smoking, %				
Never	40.4	54.0	54.2	66.0
Past[b]	55.8	25.0	35.6	18.0
Current	3.7	21.0	10.2	16.0
Median alcohol consumption, drinks/d	0.4	1.4	0.1	0.5
Mean AHEI score	41.3	35.7	48.5	39.0
Met 2008 federal physical activity guidelines[c], %	87.3	54.0	57.4	47.0
Mean BMI, kg/m^2	26.6	26.4	24.9	26.9

[a] All variables are age-adjusted based on the age distribution of the US population in 2000. HPFS = Health Professionals Follow-up Study; NHS = Nurses' Health Study; AHEI = Alternative Healthy Eating Index; BMI = body mass index

[b] Past smokers were persons who had smoked at least 100 cigarettes in their lifetime but on longer smoked at all.

[c] The 2008 federal guidelines recommend at least 150 minutes (2 hours and 30 minutes) a week of moderate-intensity or 75 minutes (1 hour and 15 minutes) a week of vigorous-intensity aerobic physical activity or an equivalent combination.

Supplementary Figures

Supplementary Figure 1. Relative Survival (%) of Common Cancers among Men Compared to the U.S. White General Population Matched on Age, Sex, Race and Calendar Year

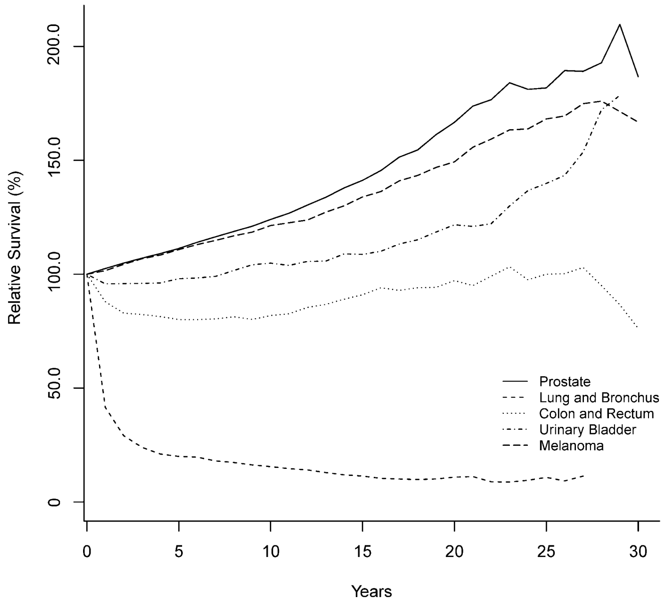

Supplementary Figure 2. Relative Survival (%) of Common Cancers among Women Compared to the U.S. White General Population Matched on Age, Sex, Race and Calendar Year

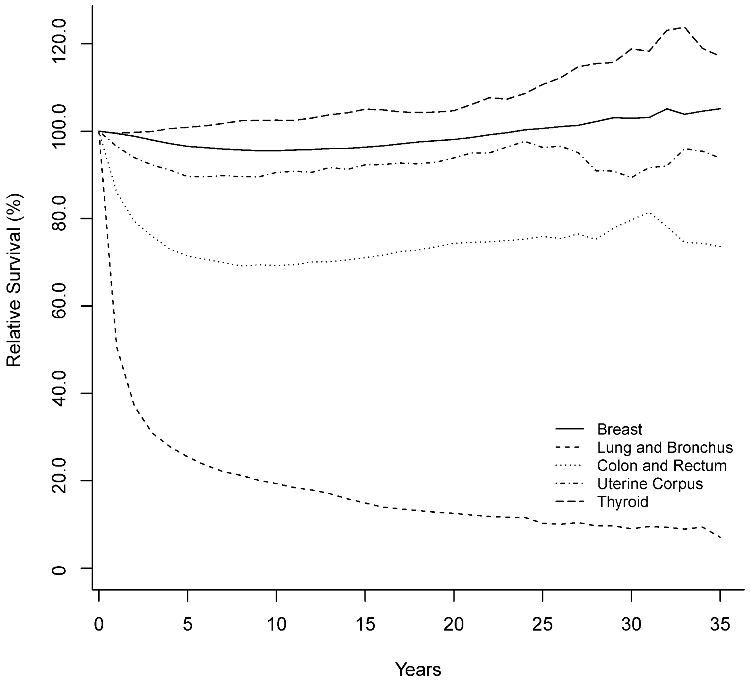

Supplementary Figure 3. Cancer-specific Cumulative Mortality (%) of Men Diagnosed with Common Cancers from SEER 18 Regs Research Database, 2000-201

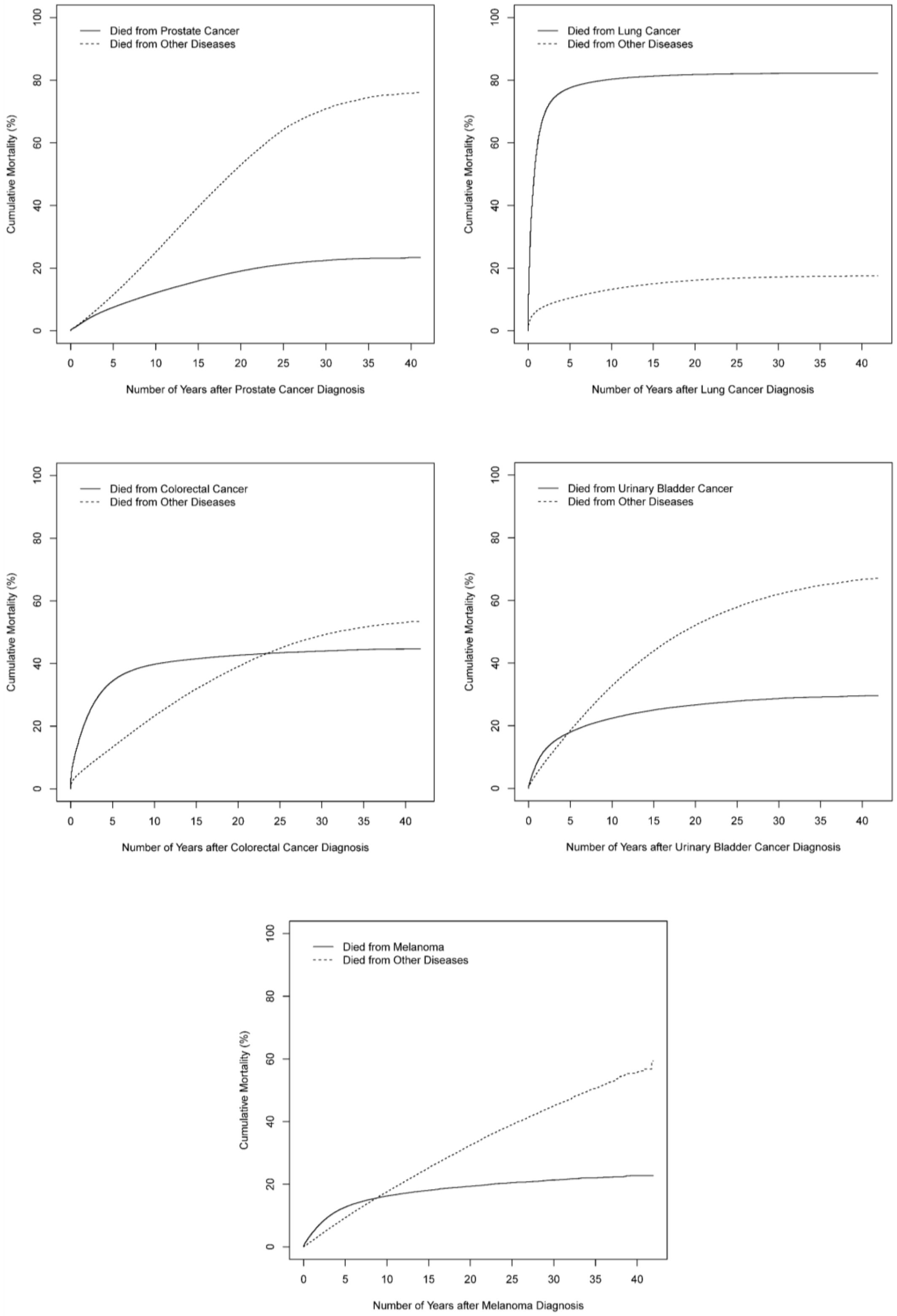

Supplementary Figure 4. Cancer-specific Cumulative Mortality (%) of Women Diagnosed with Common Cancers from SEER 18 Regs Research Database, 2000-2016

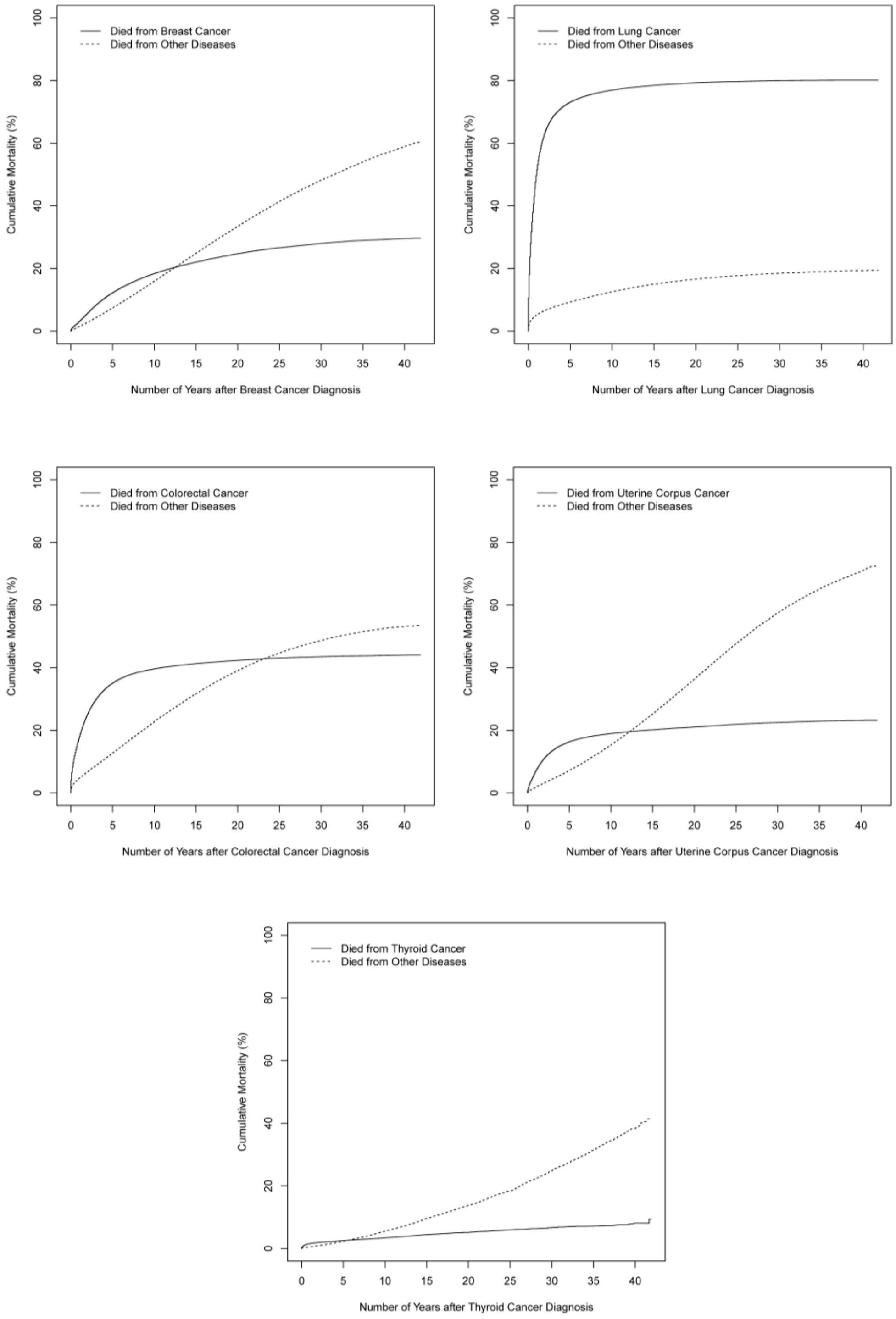

Supplement: pkac021_Supplementary_Data [file pkac021_supplementary_data.pdf]
